# The Ki67 dilemma: investigating prognostic cut-offs and reproducibility for automated Ki67 scoring in breast cancer

**DOI:** 10.1007/s10549-024-07352-4

**Published:** 2024-05-26

**Authors:** Emma Rewcastle, Ivar Skaland, Einar Gudlaugsson, Silja Kavlie Fykse, Jan P. A. Baak, Emiel A. M. Janssen

**Affiliations:** 1 Department of Chemistry, Bioscience and Environmental Engineering, University of Stavanger, Stavanger, Norway; 2 Department of Pathology, Stavanger University Hospital, Stavanger, Norway

**Keywords:** Breast cancer, Ki67, Digital image analysis, Prognostic biomarkers, Artificial intelligence

## Abstract

**Purpose:**

Quantification of Ki67 in breast cancer is a well-established prognostic and predictive marker, but inter-laboratory variability has hampered its clinical usefulness. This study compares the prognostic value and reproducibility of Ki67 scoring using four automated, digital image analysis (DIA) methods and two manual methods.

**Methods:**

The study cohort consisted of 367 patients diagnosed between 1990 and 2004, with hormone receptor positive, HER2 negative, lymph node negative breast cancer. Manual scoring of Ki67 was performed using predefined criteria. DIA Ki67 scoring was performed using QuPath and Visiopharm® platforms. Reproducibility was assessed by the intraclass correlation coefficient (ICC). ROC curve survival analysis identified optimal cutoff values in addition to recommendations by the International Ki67 Working Group and Norwegian Guidelines. Kaplan–Meier curves, log-rank test and Cox regression analysis assessed the association between Ki67 scoring and distant metastasis (DM) free survival.

**Results:**

The manual hotspot and global scoring methods showed good agreement when compared to their counterpart DIA methods (ICC > 0.780), and good to excellent agreement between different DIA hotspot scoring platforms (ICC 0.781–0.906). Different Ki67 cutoffs demonstrate significant DM-free survival (p < 0.05). DIA scoring had greater prognostic value for DM-free survival using a 14% cutoff (HR 3.054–4.077) than manual scoring (HR 2.012–2.056). The use of a single cutoff for all scoring methods affected the distribution of prediction outcomes (e.g. false positives and negatives).

**Conclusion:**

This study demonstrates that DIA scoring of Ki67 is superior to manual methods, but further study is required to standardize automated, DIA scoring and definition of a clinical cut-off.

**Supplementary Information:**

The online version contains supplementary material available at 10.1007/s10549-024-07352-4.

## Introduction

Breast Cancer is the most common cancer globally and the fifth leading cause of cancer deaths [1]. In Norway, 4,247 new cases were reported in 2022 [2]. A panel of immunohistochemistry (IHC) biomarkers can classify breast cancer into four surrogate subtypes: hormone receptors (HR) (estrogen (ER) and progesterone (PR)), HER2 and Ki67. These classify luminal A-like (low Ki67, HR+ , HER2−), luminal B-like (high Ki67, HR+ , HER2−), HER2 positive (HER2+) and triple negative (HR−, HER2−) tumors [3, 4]. The endocrine-therapy sensitive luminal-like group is the largest category and may be further categorized, using the biomarker Ki67, into luminal A-like (low-Ki67) and luminal B-like (high-Ki67). The latter benefiting from additional chemotherapy, whilst the former does not [5].

The Ki67 score defines the percentage of positively stained tumor cells in a defined hotspot or global region [6, 7] and has prognostic, predictive, and monitoring potential [8–13]. Patients with a low Ki67 score have a low recurrence risk and may be spared from chemotherapy whilst patients with a high Ki67 score are associated with an increased risk of recurrence, higher mortality rate and may benefit more from adjuvant chemotherapy [14–20].

Despite its value as a prognostic biomarker, there is no global consensus on how to standardize Ki67 scoring. This includes standardization of pre-analytical and analytical conditions, a protocol for measurement of Ki67, and cutoff score for adjuvant treatment [6, 21–24]. In 2021 the St. Gallen consensus recommended the guidelines set by the International Ki67 in Breast Cancer Working Group (IKWG): patients with low Ki67 < 5% are not recommended for adjuvant chemotherapy whilst patients with a high Ki67 ≥ 30% are recommended [21]. However, treatment recommendations for the intermediate category (> 5%, < 30%) are still debated, and other cut-offs are still used.

With the rise of digital pathology and artificial intelligence (AI), digital scoring of Ki67 by automated algorithms or applications (APPs) have provided a new avenue for improved quantification. Various studies have demonstrated equal or improved reproducibility and accuracy using automated digital image analysis (DIA) compared to manual scoring methods [9, 25–30]. However, no recommendation exists currently for automated DIA methods and established cutoffs for these methods have not yet been extensively validated.

In this study, we aim to compare the reproducibility and prognostic capacity of Ki67 score using four DIA scoring methods compared to using two manual methods (conventional-HS and global unweighted and weighted).

## Materials and methods

### Study cohort

The study received approval from the Regional Ethics Committee of Health West Norway (2010/1241) and informed consent waived. Patients who received a primary diagnosis of breast cancer between 1990 and 1998 (N = 346) and 2000–2004 (N = 253) at Stavanger University Hospital (SUH) were available for this study. Patients who received neoadjuvant treatment (N = 8) were excluded. The following inclusion criteria were used to select cases: 1) archive tumor material available as a formalin-fixed paraffin embedded (FFPE) tissue blocks from surgical excisions, 2) HR+ (ER ≥ 1%, and/or PR ≥ 10%) and HER2− status, and 3) at least one follow-up sample, for non-progression cases > 6 months after initial diagnosis. Of the 591 cases, 64 (11%) were removed due to missing blocks/lack of tumor material, 19 (3%) were lost to follow-up and 141 HER2+ /TNBC were excluded. In summary, 367 cases comprised the study cohort.

### Datasets

The development dataset consisted of a training and tuning set (Fig. S1). A training set was used to create manual annotations to train classification algorithms. Five whole slide images (WSI) of good quality and representative of strong and weak staining, and high and low Ki67 positivity were selected. The WSIs were annotated with training labels for segmentation of key features: tissue and background, tumor and non-tumor, and positive brown and negative blue nuclei. Two of the four DIA methods required training (VIS1-HS, QuPath), whilst the two remaining methods (VIS2-HS, VIS2-G) were pre-trained and validated. All DIA methods were run on the tuning dataset, which was used to monitor and evaluate algorithm performance, assess reproducibility, and define prognostic cutoffs.

### Immunohistochemistry and imaging

New 3 µm tissue sections were cut, mounted on SuperFrost® Plus slides (Menzel Gläser, Braunschweig, Germany), and dried overnight at 37 °C followed by 1 h at 60 °C. Sections were transferred deparaffinized to the Dako Omnis (Dako, Glostrup, Denmark). Antigen retrieval was performed using the EnVision FLEX Target Retrieval Solution High pH (Dako Omnis), heated at 97 °C for 30 min. Sections were stained for Ki67 using a pre-optimized protocol (diagnostic protocol). Given the age of the tissue blocks, the diagnostic protocol required adjustment to address the issue of false negative staining. The Dako MIB-1 clone was diluted 1:50 (diagnostic protocol uses 1:100) and incubated for 20 min. Additionally, signal amplification was performed using the EnVision FLEX + Mouse LINKER (Dako Omnis) with a 10-min incubation (diagnostic protocol uses FLEX). Previously stained slides from 2011 were matched and compared to the newly stained sections, using the adjusted protocol, to ensure equivalent results.

### Manual hotspot Ki67 quantification (conventional)

Using a microscope, the whole section was viewed at low power to identify the most proliferative region (hotspot) in the invasive tumor region. Non-invasive regions, necrotic regions and areas with high lymphocytic infiltration were avoided. Two manual methods, which reflected the diagnostic setting at the time of counting, were used for the 1990–1998 and 2000–2004 cohorts. Manual counts were performed previously and available in the pre-established database.

For the 1990–1998 cohort, a 40X objective was used to count positively stained tumor nuclei (brown) and the total number of tumor nuclei in the hotspot. For each case, at least 500 tumor cells were counted. If fewer than 500 tumor cells, adjacent fields of view (FOV) were counted. Ki67 score was calculated as the percentage positive Ki67 (Ki67 positive/(Ki67 positive + Ki67 negative)).

For the 2000–2004 cohort, the interactive QPRODIT system (Leica, Cambridge, UK) was used to score Ki67 as described previously [31]. Within a hotspot tumor region, 250–350 FOV were defined, and a test grid used to classify each field as Ki67 positive or negative. A field was classified as positive if the first tumor cell that intersected with a grid point was positive, and vice versa for negative cells. Ki67 score was calculated as the percentage positive Ki67.

Both methods were grouped under the term conventional Ki67 score. No significant differences were observed between the two cohorts (Table S1).

### Manual global Ki67 quantification (global unweighted and weighted)

Global weighted and unweighted scoring was performed according to the protocol set by the IKWG [32]. The IKWG Ki67 mobile counting tool was used (https://www.ki67inbreastcancerwg.org/). Using the NDP2.view2 image viewing software (v.2.9.29, Hamamatsu Photonics, Japan), a WSI of the Ki67-stained tissue was examined and the percentage area of negligible, low, medium, or high Ki67 was estimated and entered into the counting tool. Three to four circular annotations were placed to simulate a field of view, in each field type, as directed by the tool. In a typewriter fashion, 100 nuclei were counted as either negative or positive in each ROI. The unweighted and weighted global scores were recorded for each slide.

### Digital image analysis (DIA)

#### Scanning

Whole sections stained with Ki67 were scanned at 40X magnification using the Hamamatsu Nanozoomer S60 (Hamamatsu Photonics, Hamamatsu City, Japan) at SUH.

Two platforms were used to score Ki67 on whole slide images (WSI): QuPath [33] and Visiopharm® (Version 2022.09.3.12885, Visiopharm A/S, Hørsholm, Denmark). The following hardware was used: Dell Precision 3640 Tower, Intel Core i9-10900, Nvidia GeForce RTX 2080 Ti.

#### VIS1-HS

**Fig. 1:**
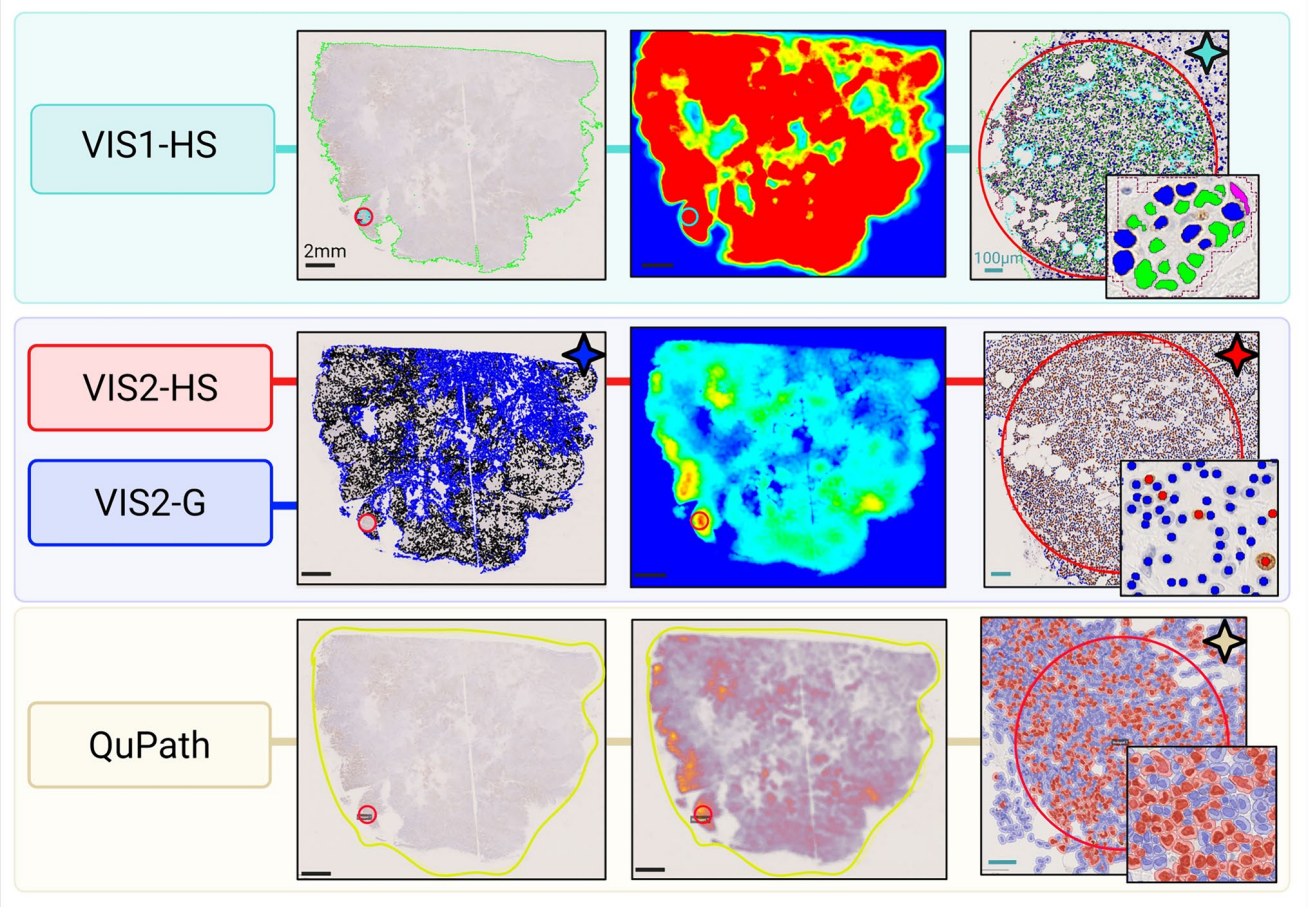
Visualization of each digital image analysis Ki67 scoring method. The VIS1-HS method, developed in-house using the Visiopharm® commercial platform, utilized a 1mm^2^ hotspot (red/cyan ROI) for Ki67 scoring. The VIS2 method, a commercial application provided by Visiopharm®, measured both global Ki67 score in the entire tumor region—blue ROI (VIS2-G) and in a 0.96mm^2^ hotspot—red ROI (VIS2-HS). The QuPath method required manual demarcation of the tissue (yellow ROI). The Ki67 score was calculated in a hotspot (red ROI) with minimum 500 tumor cells. Stars indicate when the Ki67 score was calculated for each method. *ROI* region of interest*, VIS1-HS* Visiopharm in-house hotspot, *VIS2-HS* Visiopharm CE-IVD hotspot, *VIS2-G* Visiopharm CE-IVD global

An in-house APP (VIS1-HS) was developed using the Visiopharm® platform for quantification of Ki67 score (Fig. 1). Six standalone APPs were developed, that were batch run: 01 Tissue detection, 02 Positive nuclei detection, 03 Hotspot detection; 04 Tumor detection, 05 Nuclei segmentation, and 06 Hotspot Ki67 score quantification. Manual annotations from the training dataset were used to create labels to train the classifier APPs: (01) tissue and non-tissue training labels to train a tissue classifier, (02) positive nuclei labels to train a positive nuclei classifier, (04) tumor and non-tumor labels to train a tumor classifier, and (05) positive and negative nuclei labels to train a nuclei classifier. Training was performed until the classifier achieved accurate and consistent predictions, as determined by the operator. All classifier APPs used supervised K-means clustering. Specific parameters were trialed with the VIS1-HS APP (Fig. S1): the drawing radius for the heatmap configuration (175 µm, 400 µm), number of hotspot ROI (1–5), and ROI size criteria (0.2mm^2^, 1mm^2^, minimum 550 tumor cell count, minimum 1000 tumor cell count).

*VIS2-HS/G:* Visiopharm® provided a commercial, CE-IVD Ki67 quantification APP (VIS2-HS/G) (Fig. 1). This method consists of six standalone APPs that were batch run, in the following order: 01 #10,182—IHC Tissue Detection, AI; 02 #10180—Invasive Tumor Detection, AI; 03 #10180—Invasive Tumor Postprocessing; 04 #10173—Ki-67 Nuclei APP, Breast Cancer, AI; 05 #10114 Hot Spot Detection; 06 #10114 Hot Spot Quantification (Table S2). This method generated both a global score (VIS2-G), which assessed the invasive tumor region of the entire WSI, and a hotspot score (VIS2-HS).

#### QuPath

A classifier for quantification of Ki67 score was developed using QuPath (Fig. 1), based on the protocol established by Acs and colleagues [34]. To train the cell classifier, positive and negative tumor cell nuclei were annotated manually on WSI from the training dataset. A tumor ROI was first manually drawn around the invasive tumor region and the cell classifier script (Table S3) run. The density maps tool generated a heatmap and three ROIs were automatically placed on suspected hotspots. Each hotspot was reviewed, and the highest scoring hotspot was approved according to the following criterion: minimum 500 tumor cells and satisfactory labelling of positive and negative tumor nuclei. If the ROI contained less than 500 tumor cells, it was manually enlarged until this criterion was met. The QuPath method was semi-automated; manual delineation of the tumor region was required for each case, and the classifier and density maps were run separately per case.

### Statistical analysis

Statistical analyses were performed with SPSS for Windows (version 26.0.0; IBM SPSS Statistics) and R studio (2023.06 + 561). Assumptions were verified for each test and p < 0.05 was significant. Mann Whitney U and Kruskall Wallis tests were used to test for significant differences between patients with no distant metastases (DM) and with DM. Level of agreement, using the intraclass correlation coefficient (ICC), was assessed on transformed Ki67 scores (multiplied by a factor of 10 and log transformed). Receiving operating characteristic (ROC) curves were generated for each scoring method and the area under the curve (AUC) was used to assess a method’s discriminative ability. Kaplan Meier survival analyses were performed to assess the prognostic value of DM-free survival and compared using log-rank (endpoint: first diagnosis of a DM in the follow-up or censored according to last-known follow-up date). Cox regression models for univariate and multivariate analysis was performed for: age, tumor size, mitotic activity index, Nottingham grade, operation type, adjuvant treatment, and Ki67 score.

## Results

### Overview

Of the 367 cases from the development dataset, 12 cases were ineligible for analysis due to poor quality material and 61 cases due to poor staining (Fig. S2). This left 294 cases (Table S4). A further 9 cases failed analysis with QuPath due to: a false positive edge effect, high numbers of inflammatory cells, necrosis, and artefacts (Fig. S2).

### Performance of DIA methods

For the VIS1-HS method various ROI specifications were trialed and differences in Ki67 scores for each was considered marginal (Table S5). A 1mm^2^ ROI was selected for the final VIS1-HS method as it was the most consistent for detecting over 500 tumor cells.

All DIA methods required manual editing for some or all cases. For VIS1-HS, nearly all cases required manual removal of either DCIS, artefacts, inflammatory cell clusters or normal tissue. For VIS2-HS, 13% of cases (37/294) required a manual edit. The VIS2-G method required a manual edit in 28% of cases (81/294). For QuPath, all cases required a manual delineation of the tissue region for analysis, and 45% of cases (128/285) required a manual intervention for HS ROI placement or expansion.

### Comparison of Ki67 score for four DIA scoring and two manual methods

For DIA and manual scoring methods the mean Ki67 score ranged from 9.5% (VIS2-G) to 16.2% (QuPath) (Table 1, Fig. S3). For cases with no distant metastases (no DM) compared to cases with distant metastases (DM) in the follow-up, the greatest mean difference was recorded for QuPath (11.9%), followed by the VIS2-HS (9.6%), global weighted (8.6%), VIS1-HS (7.2%), VIS2-G (6.9%), global unweighted (6.8%), and conventional hotspot (5.9%) methods. Furthermore, QuPath recorded higher scores on average, with the VIS2-G method recording the lowest (Table 1).

**Table 1:** Quantification of Ki67 and total tumor cell count according to four digital scoring methods, and two manual scoring methods (conventional hotspot, global unweighted and weighted)

Method	N =	%Ki67 (all)	%Ki67 (No DM)	%Ki67 (DM)	Total tumor cell count (all)
Conventional hotspot	N = 284 No DM: 243 DM: 41	13.7 (0–83)	12.8 (0–83)	18.7 (0–65)	No data
Global unweighted	N = 294 No DM: 254 DM: 40	15.9 (0–100)	15.0 (0–100)	21.8 (0–57.3)	No data
Global weighted	N = 294 No DM: 254 DM: 40	16.0 (0–100)	14.9 (0–100)	23.5 (0–77.9)	No data
VIS1-HS	N = 294 No DM: 254 DM: 40	13.3 (0–79)	12.3 (0–79)	19.5 (2.8–74.6)	2907 (410–12727)^a^
VIS2-HS	N = 294 No DM: 254 DM: 40	14.4 (0.2–89.8)	13.1 (0.2–.78.2)	22.7 (1.1–89.8)	4094 (251–11853)^b^
VIS2-G	N = 294 No DM: 254 DM: 40	9.5 (0.1–70.3)	8.6 (0.1–.70.3)	15.5 (0.7–64.6)	122966 (531–1013920)
QuPath	N = 285 No DM: 245 DM:40	16.2 (0–92.9)	14.6 (0–91.3)	26.5 (2.5–92.9)	662 (500–1697)

Mean values are reported with range in parentheses

*No DM* no distant metastasis, *DM* distant metastasis, *VIS1-HS* Visiopharm in-house hotspot, *VIS2-HS* Visiopharm CE-IVD hotspot, *VIS2-G* Visiopharm CE-IVD global

^a^Two cases were < 500 tumor cells

^b^Three cases were < 500 tumor cells

Reproducibility is one of the primary concerns regarding scoring of Ki67. In the study cohort, there was moderate to excellent agreement between all scoring methods (Table 2). Comparison of all hotspot scoring methods revealed good to excellent agreement (ICC 0.781–0.906). Agreement was on average higher between automated hotspot methods than between automated and manual hotspot methods (Table 2). There was good agreement between the global DIA method and manual global method (ICC 0.803–0.810). Agreement was lower between global and hotspot scoring methods (ICC 0.636–0.759).

**Table 2:** Agreement of manual and digital image analysis Ki67 quantification methods as assessed by the intraclass correlation coefficient (ICC)

Method	Measure	Method					
		Conventional	Global unweighted	Global weighted	VIS1-HS	VIS2-HS	VIS2-G
Conventional	ICC (95% CI)						
Global unweighted	ICC (95% CI)	0.758 (0.698–0.806)					
Global weighted	ICC (95% CI)	0.746 (0.684–0.797)	0.986 (0.982–0.989)				
VIS1-HS	ICC (95% CI)	0.802 (0.743–0.848)	0.648 (0.559–0.719)	0.636 (0.535–0.715)			
VIS2-HS	ICC (95% CI)	0.846 (0.794–0.884)	0.759 (0.698–0.807)	0.752 (0.681–0.806)	0.874 (0.843–0.899)		
VIS2-G	ICC (95% CI)	0.834 (0.642–0.909)	0.803 (0.731–0.853)	0.810 (0.756–0.852)	0.735 (0.166–0.887)	0.863 (0.152–0.955)	
QuPath	ICC (95% CI)	0.781 (0.680–0.845)	0.729 (0.643–0.792)	0.709 (0.609–0.781)	0.835 (0.796–0.867)	0.906 (0.881–0.926)	0.786 (0.153–0.918)

*VIS1-HS* Visiopharm in-house hotspot, *VIS2-HS* Visiopharm CE-IVD hotspot, *VIS2-G* Visiopharm CE-IVD global, *ICC* intraclass correlation coefficient, *CI confidence intervals*

### Defining a prognostic threshold

A ROC curve was generated to assess DM-free survival and identify optimal cutoffs. The two methods with the highest AUC were QuPath, 0.721 (95% CI 0.643–0.798) and VIS2-HS, 0.705 (95% CI 0.625–0.785). The manual methods recorded the lowest AUC: conventional hotspot, 0.655 (95% CI 0.569–0.741); global weighted, 0.648 (95% CI 0.554–0.744); and global unweighted, 0.636 (95% CI 0.541–0.731). A range of coordinates were selected from the ROC curve for binary categorization of Ki67 score, which revealed similar DM-free survival (Table S6, Fig. S4). The manual methods demonstrated lower p-values (log-rank) for cut-offs around 10%, whereas automated methods reported lower p-values between 10 and 14% (Table S6). A 14% cutoff was chosen for further evaluation, due to its recommendation by the Norwegian Guidelines [35].

**Fig. 2:**
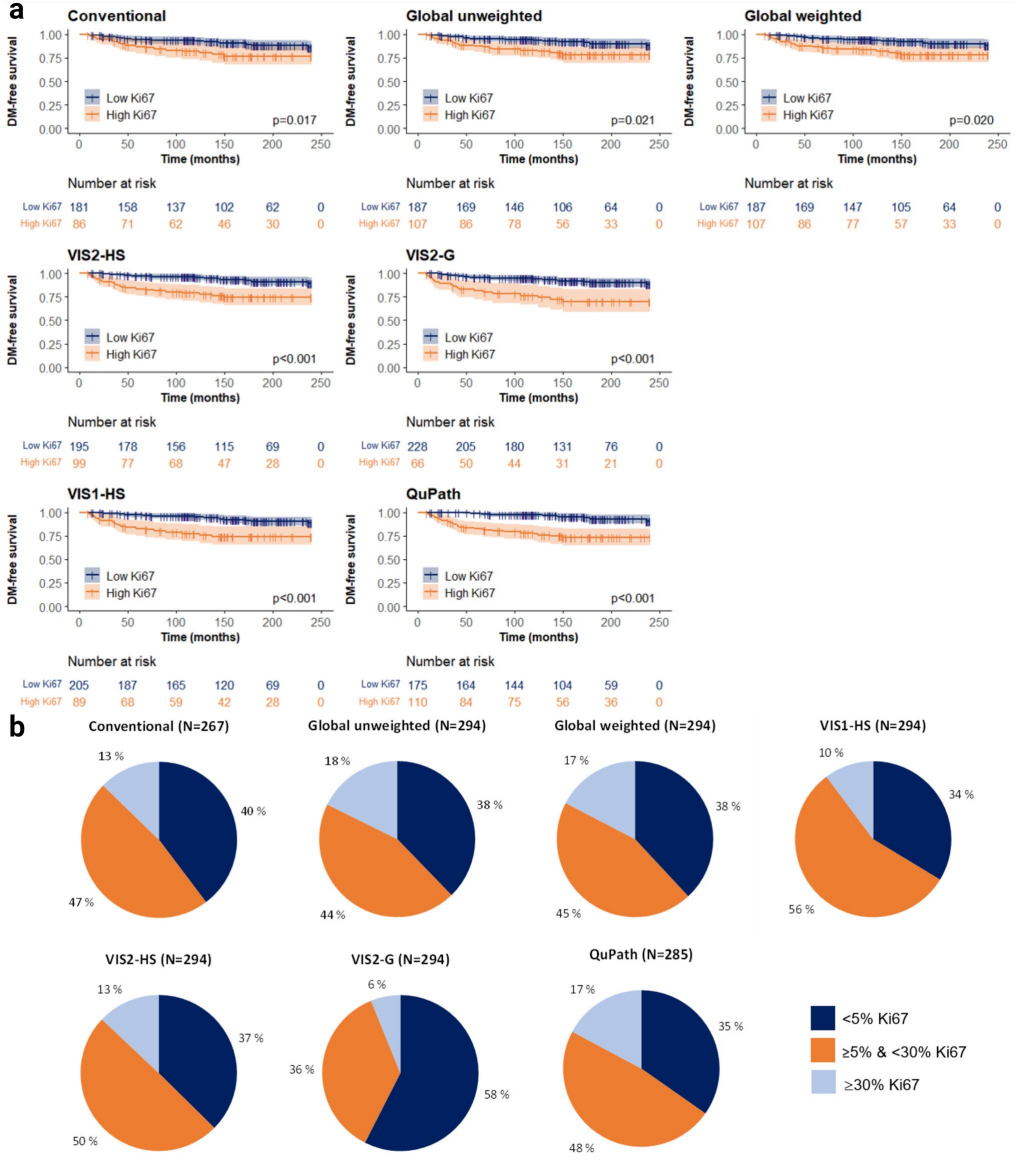
Evaluation of a prognostic Ki67 cutoff in breast cancer. **a** Distant-metastasis free survival of patients with HR+ , HER2−, LN− breast cancer categorized by low Ki67 (< 14%) and high Ki67 (≥ 14%) over a 20-year follow-up period. Scoring of Ki67 was performed by two manual scoring methods (conventional hotspot and global weighted and unweighted) and four digital image analysis methods: VIS1-HS, QuPath, VIS2-HS and VIS2-G. **b** Proportion of cases assigned a low (< 5%), intermediate (≥ 5% & < 30%) and high (≥ 30%) Ki67 score according to two manual scoring methods (conventional hotspot, global weighted and unweighted) and four DIA scoring methods (VIS1-HS, VIS2-HS, VIS2-G, and QuPath). *VIS1-HS* Visiopharm in-house hotspot*, VIS2-HS* Visiopharm CE-IVD hotspot*, VIS2-G* Visiopharm CE-IVD global

Binary categorization of Ki67-14% for all methods was significantly associated with DM-free survival in a 20-year follow-up period (Fig. 2a). All methods demonstrated a significant separation of patients with DM-free survival for low (< 14%) and high (≥ 14%) Ki67, with the manual methods reporting the smallest separation in comparison to DIA (Fig. 2a). Additionally, percentage agreement was highest for VIS1-HS and VIS2-HS methods and lowest between VIS2-G and QuPath methods (Table S7).

**Fig. 3:**
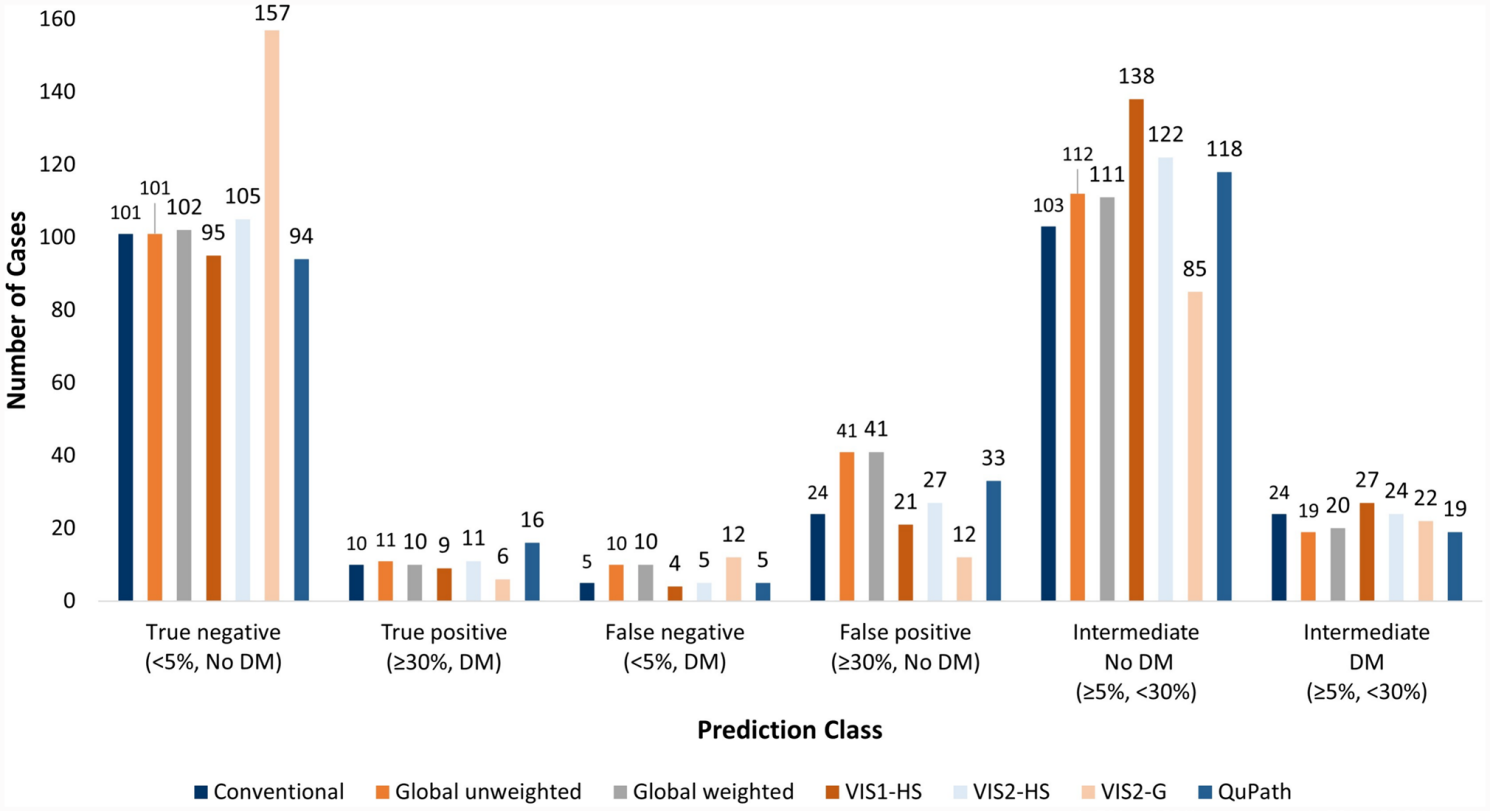
Prediction classes using < 5% and ≥ 30% Ki67 thresholds set by the International Ki67 in Breast Cancer Working Group, as measured by a manual and digital image analysis scoring methods. Prediction classes are assigned by %Ki67 and absence of distant metastases (no DM) or presence of distant metastases in the follow-up (DM). *VIS1-HS* Visiopharm in-house hotspot*, VIS2-HS* Visiopharm CE-IVD hotspot*, VIS2-G* Visiopharm CE-IVD global

International recommendations suggest < 5% to assign low Ki67 and ≥ 30% to assign high Ki67, with remaining cases falling into an intermediate category. The VIS2-G method had the largest proportion of low (< 5%) Ki67 cases (58%) of all methods (Fig. 2b). QuPath and global weighted/unweighted methods had the highest proportion of high (≥ 30%) Ki67 cases (17–18%). The VIS2-G method reported the highest number of false negatives (low Ki67, DM), whilst the highest number of false positives was recorded by the manual global methods (high Ki67, no DM), respectively (Fig. 3). This was also observed at a 10-year follow-up.

### Multivariate analysis

To assess the prognostic value of Ki67 scoring, we performed Cox regression analysis of each method alongside established prognostic markers. Of the variables tested, Nottingham grade, mitotic activity index (MAI10), tumor-size (2 cm), operation type, and Ki67 for all methods (14%) were statistically significant predictors of DM-free survival in univariate analysis (Table 3). Hazard ratios (univariate) for all DIA methods (Ki67 14%) ranged from 3.054 to 4.077, with overlapping 95% confidence intervals whilst the manual methods had lower hazard ratios: 2.012–2.056 (Table 3). In a multivariate analysis, Nottingham Grade, adjuvant treatment, and operation type were predictors in the final model.

**Table 3:** Distant-metastasis free survival in hormone receptor positive, HER2 negative, lymph node negative, breast cancer patients (values reported in italics use continuous variables*)*

Characteristic	All	(%)	Log rank p-value	Hazard Ratio	Cox regression p-value	95% CI
	Event/At risk					
Age	N = 316			*0.976*	*0.168*	*0.944–1.010*
< 55	18/134	13	0.676	1.140	0.677	0.617–2.107
≥ 55	24/182	13				
Nottingham Grade	N = 314					
Grade I (3–5)	6/108	6	0.001*	2.441	0.054	0.984–6.053
Grade II (6–7)	21/149	14		5.344	< 0.001*	2.070–13.794
Grade III (8–9)	15/57	26				
Tumor size^a^	N = 314			*1.927*	< 0.001*	*1.404–2.645*
≤ 2 cm	28/258	11	0.003*	2.553	0.004*	1.341–4.862
> 2 cm	14/56	25				
Operation Type	N = 250					
Conservative/Lumpectomy	15/152	10	0.004*	2.524	0.005*	1.313–4.850
Mastectomy	23/98	23				
MAI	N = 309			*1.018*	*0.062*	*0.999–1.037*
< 10	26/242	11	0.005*	2.410	0.007*	1.276–4.553
≥ 10	15/67	22				
Ki67						
Conventional	N = 267			*1.014*	0.041*	*1.001–1.028*
< 14	20/181	11	0.026*	2.012	0.029*	1.073–3.774
≥ 14	19/86	22				
Global unweighted	N = 294			*1.010*	*0.133*	*0.997–1.023*
< 14	19/187	10	0.021*	2.047	0.024*	1.099–3.813
≥ 14	21/107	20				
Global weighted	N = 294			*1.012*	0.048*	*1.000–1.024*
< 14	19/187	10	0.020*	2.056	0.023*	1.104–3.829
≥ 14	21/107	20				
VIS1-HS	N = 294			*1.021*	0.006*	*1.006–1.037*
< 14	18/205	9	< 0.001*	3.054	< 0.001*	1.633–5.711
≥ 14	22/89	25				
VIS2-HS	N = 294			*1.023*	0.001*	*1.010–1.037*
< 14	16/195	8	< 0.001*	3.245	< 0.001*	1.721–6.116
≥ 14	24/99	24				
VIS2-G	N = 294			*1.025*	0.004*	*1.008–1.042*
< 14	22/228	10	< 0.001*	3.088	< 0.001*	1.654–5.766
≥ 14	18/66	27				
QuPath	N = 285			*1.022*	< 0.001*	*1.010–1.034*
< 14	12/175	7	< 0.001*	4.077	< 0.001*	2.070–8.030
≥ 14	28/110	25				

*CI* confidence intervals*, MAI* mitotic activity index*, VIS1-HS* Visiopharm in-house hotspot*, VIS2-HS* Visiopharm CE-IVD hotspot*, VIS2-G* Visiopharm CE-IVD global

*Denotes significance p < 0.05

^a^Only one case had a tumor size ≥ 5 cm, so tumor size was grouped into ≤ 2 cm and > 2 cm (T1 and T2), instead of three groups (T1, T2, T3)

## Discussion

We compare several automated DIA tools for global and hotspot Ki67 to two manual methods in HR+ , HER2−, LN− breast tumors. Although Ki67 is considered an important biomarker in breast cancer, the concerns surrounding lack of standardization and poor reproducibility, have brought its value into question. In this study, we observed that commercial DIA tools (VIS2-HS, VIS2-G) required notably less manual editing compared to the in-house methods (VIS1-HS, QuPath).

Inter-platform variability demonstrated good to excellent agreement between all hotspot scoring methods and all global methods (ICC > 0.8). Another inter-platform study reported excellent reproducibility (ICC > 0.9) [26] and our observation of strong agreement between manual and DIA platforms is consistent with observations in the literature [7, 9, 36–42]

Although efforts have been made to standardize Ki67 scoring, both hotspot and global Ki67 score are still reported in the literature and in Norway, Sweden, and Denmark [6, 38, 43, 44]. In addition, a range of cutoffs for defining low and high Ki67 are still reported (range: 10–20%) [17, 26, 45, 46]. We observed a range of prognostic cutoffs, with 14% being optimal and in agreement with Norwegian guidelines [6]. The hazard ratios (HR 2.7–3.7) reported by Acs et al. [26] for DIA scoring on core needle biopsies and tissue microarrays were similar to those reported in our study (HR 3.1–4.1) and Boyaci et al. [47] (HR 2.6–4.2) for DIA scoring on surgical specimens. Furthermore, we observed that DIA methods had a greater discriminative capacity, using the AUC metric, than manual methods. This was reflected in the hazard ratios for Ki67 score (14%) and DM-free survival (DIA HR 3.054–4.077 vs. Manual HR 2.012–2.056). Another study observed similar hazard ratios for DIA scoring (hotspot HR: 6.88; global HR 3.13) compared to a manual hotspot method (HR 2.76), for recurrence free survival [48].

In 2021, the St. Gallen consensus adopted the IKWG recommendation of < 5% (low) and > 30% (high), with patients between 5 and 30% (intermediate) not recommended for treatment decisions by Ki67 [38]. In our study, evaluation of Ki67 score using these thresholds revealed that QuPath had the largest proportion of Ki67 high cases and highest number of false positives (high Ki67, no DM). Whilst, the VIS2-G, global scoring method, demonstrated the highest proportion of Ki67 low cases and highest number of false negatives (low Ki67, DM). This suggests that regardless of using a more restrictive cutoff, patients are still at risk of over- and under-treatment. Furthermore, assessing the number of false positives and negatives revealed differences in clinical consequence between methods. This is important for future implementation of DIA methods as the choice of method: hotspot or global scoring, can have notable differences in classification of patients.

A high total tumor count resulted in lower Ki67 scores. The average total number of tumor cells measured for global DIA score was > 100,000, far more than for all other methods and it consistently reported lower Ki67 scores than the others. Norwegian guidelines and IKWG recommendations for Ki67 scoring recommend a minimum of 400 to 500 tumor cells scored [6, 49]. Our results suggest that Ki67 scores from tumor cell counts around 2000–4000 cells, generated by a 1mm^2^ ROI, were more consistent, with fewer potentially under- or over-treated cases. Observations from Robertson et al. revealed greater reproducibility with increasing tumor cell counts (from 200 to 1000 tumor cells) [48]. This suggests the need for caution when translating current manual methods to a DIA method. A larger study is required to affirm the optimal number of total tumor cells for Ki67 score by DIA.

As use of molecular signature tests for classification of breast cancer increases, the use of Ki67 for treatment decisions is called into question. Molecular testing has demonstrated prognostic and predictive value [50–54]. However, where such molecular panels are unavailable, considered costly, or introduce delays to treatment due to slower return of results, Ki67 could be an equivalent approach. Furthermore, Ki67 has the potential to be used as a screening tool for recommending molecular testing in intermediate cases (Ki67 > 5%, < 30%) [38]. Majority consensus warrants use of both multigene panels and Ki67 score [21].

This study does not come without its limitations. The study utilized a retrospective cohort, and many of the tissue blocks were > 20 years old, therefore the staining protocol had to be adjusted due to antigen decay. Additionally, only one scanner type and one automated IHC-instrument was used whereas multiple different scanners and staining methods, from different locations and time periods, on different or the same tissue blocks, would be worth investigating. In the present study, we only used one global scoring DIA method (VIS2-G), and future work to compare global DIA reproducibility could be pursued. For a future study, it would be worthwhile to investigate DIA scoring of Ki67 in a prospective cohort, with molecular profile data, with a planned long-term follow-up, such as the EMIT study [55].

In summary, we report good agreement between manual and counterpart DIA scoring methods. DIA Ki67 scoring methods had a greater discriminative capacity for DM-free survival than manual methods. A range of cutoffs was prognostic for each method, but the choice of scoring method and cutoff can lead to notable differences in the number of patients to be treated or tested, emphasizing the need for further validation in a prospective cohort. Total tumor cell count contributed to changes in risk categorization using the recommended 5% and 30% threshold. Automated, DIA methods may improve reproducibility and prognostic value of Ki67 scoring in comparison to manual methods, if standardized.

### Supplementary Information

Below is the link to the electronic supplementary material.

Supplementary file1 (PDF 955 KB)

## Data Availability

The patient databases used in this study are not publicly available due to ethical and legal concerns. Anonymized data can be requested from Stavanger University Hospital Institutional Data Access/Ethics Committee (contact via email: rek-vest@uib.no, REK vest, Rogaland, Vestland, Norway) for researchers who meet the criteria for access to confidential data.
