# Supplementary material for: The Ki67 dilemma: investigating prognostic cut-offs and reproducibility for automated Ki67 scoring in breast cancer
Source: Breast Cancer Res Treat. 2024 May 26;207(1):1–12. doi: 10.1007/s10549-024-07352-4 (PMC11231004; doi:10.1007/s10549-024-07352-4)
Supplement: Supplementary file 1 — Supplementary file1 (PDF 955 KB) [file 10549_2024_7352_MOESM1_ESM.pdf]

Emma Rewcastle[a,b], Ivar Skaland[b], Einar Gudlaugsson[b], Silja Kavlie Fykse[b], Jan P.A. Baak[b], Emiel A.M. Janssen[a,b]*
[a]Department of Chemistry, Bioscience and Environmental Engineering, University of Stavanger, Stavanger, Norway. [b]Department of Pathology, Stavanger University Hospital, Stavanger, Norway.
**Corresponding author. E-mail address: emma.rewcastle@sus.no.*
Breast Cancer Research and Treatment

Supplementary Information

Supplementary Table S1

Supplementary Table S1: Comparison of differences between the 1990-1998 and 2000-2004 cohort for the variables age, mitotic activity index, Nottingham grade and Ki67 manual hotspot score.

Variable	Descriptives				Statistical test
	1990-1998 cohort		2000-2004 cohort		
N=	190 (189[a])		77		n/a
Age	Mean<br>Median<br>Range	56<br>57<br>29-70	Mean<br>Median<br>Range	55<br>55<br>32-70	Mann-Whitney U<br>*U*= 6928.5, p=0.499
Mitotic activity index[a]	Mean<br>Median<br>Range	7<br>3<br>0-73	Mean<br>Median<br>Range	8<br>3<br>0-87	Mann-Whitney U<br>*U*= 7110.0, p=0.768
Tumor Size	Mean<br>Median<br>Range	1.55<br>1.50<br>0.20-3.50	Mean<br>Median<br>Range	1.56<br>1.40<br>0.6-6.5	Mann-Whitney U<br>*U*= 6694.0, p=0.276
Nottingham Grade	I<br>II<br>III	66 (35%)<br>90 (47%)<br>34 (18%)	I<br>II<br>III	23 (30%)<br>37 (48%)<br>17 (22%)	Pearson Chi-Square<br>χ^2 = 0.897, p=0.639
Ki67 (manual hotspot)	Mean<br>Median<br>Range	13.3%<br>7%<br>0-83%	Mean<br>Median<br>Range	14.7%<br>7%<br>0-82%	Mann-Whitney U<br>*U*= 6867.5, p=0.433

Abbreviations: n/a = not applicable

Supplementary Table S2

Supplementary table S2: Identification and version number for each Visiopharm® AI Ki67 Application

Number	Identification	Version
01	#10182 – IHC Tissue Detection	2022.09.0.12236
02	#10180 – Invasive Tumor Detection	2022.07.0.12224
03	#10180 – Invasive Tumor Postprocessing	2022.07.0.11960
04	#10173 – Ki-67 Nuclei APP, Breast Cancer, AI	2022.07.0.11960
05	#10114 Hot Spot Detection	2022.09.0.12417
06	#10114 Hot Spot Quantification	2022.09.0.12417

Supplementary Table S3

Supplementary table S3: The Ki67 nuclei classifier script developed in QuPath.

setImageType('BRIGHTFIELD_H_DAB');
setColorDeconvolutionStains('{"Name" : "H-DAB default", "Stain 1" : "Hematoxylin", "Values 1" : "0.65111 0.70119 0.29049 ", "Stain 2" : "DAB", "Values 2" : "0.26917 0.56824 0.77759 ", "Background" : " 255 255 255 "}');
runPlugin('qupath.imagej.detect.cells.PositiveCellDetection', '{"detectionImageBrightfield": "Optical density sum", "requestedPixelSizeMicrons": 1.0, "backgroundRadiusMicrons": 10.0, "medianRadiusMicrons": 0.1, "sigmaMicrons": 1.5, "minAreaMicrons": 15.0, "maxAreaMicrons": 400.0, "threshold": 0.1, "maxBackground": 2.0, "watershedPostProcess": true, "excludeDAB": false, "cellExpansionMicrons": 5.0, "includeNuclei": true, "smoothBoundaries": true, "makeMeasurements": true, "thresholdCompartment": "Nucleus: DAB OD mean", "thresholdPositive1": 0.2, "thresholdPositive2": 0.4, "thresholdPositive3": 0.6000000000000001, "singleThreshold": true}');
runPlugin('qupath.lib.plugins.objects.SmoothFeaturesPlugin', '{"fwhmMicrons": 25.0, "smoothWithinClasses": false}');
runPlugin('qupath.lib.plugins.objects.SmoothFeaturesPlugin', '{"fwhmMicrons": 25.0, "smoothWithinClasses": true}');
runPlugin('qupath.lib.plugins.objects.SmoothFeaturesPlugin', '{"fwhmMicrons": 25.0, "smoothWithinClasses": false}');
runPlugin('qupath.lib.plugins.objects.SmoothFeaturesPlugin', '{"fwhmMicrons": 25.0, "smoothWithinClasses": false}');

Supplementary Table S4

Supplementary Table S4: Overview of patient characteristics including age, follow-up time, tumor size, Nottingham Grade, treatment, and operation type for the 294 cases eligible for conventional and digital analysis of Ki67.

**Characteristics**	**N=**				**All**	**No DM**	**DM**
	All	*No DM*	*DM*				
**Age** Mean (Range)	294	254	40		56 (29-70)	56 (32-70)	53 (29-70)
**Follow-up time (months)*** Mean (range)	294	254	40		161 (8-345)	173 (9-345)	88 (8-302)
**Tumor size****	292	252	40		1.55 (0.2-6.5)	1.49 (0.2-4.2)	1.92 (0.7-6.5)
**Nottingham Grade*****	292	252	40	I	102 (35%)	96 (38%)	6 (15%)
				II	138 (47%)	118 (47%)	20 (50%)
				III	52 (18%)	38 (15%)	14 (35%)
**Adjuvant Systemic Therapy**	202	167	35	NTR	187 (93%)	158 (95%)	29 (83%)
				TR[a]	15 (7%)	9 (26%)	6 (17%)
**Operation Type******	231	195	36	Conservative/ Lumpectomy	139 (60%)	124 (64%)	15 (42%)
				Mastectomy	92 (40%)	71 (36%)	21 (57%)

Abbreviations: DM = distant metastasis, NTR = no treatment, TR = treatment

**Significant difference between no DM and DM groups (Mann-Whitney U, p<0.001)*

***Significant difference between no DM and DM groups (Mann-Whitney U, p=0.007)*

****Significant difference between no DM and DM groups (Kruskall Wallis, p<0.001)*

*****Significant difference between no DM and DM groups (Kruskall Wallis, p=0.014)*

[a]Treatment types included chemotherapy, HRT or chemotherapy combined with HRT

Supplementary Table S5

Supplementary table S5: Overview of the region of interest (ROI) specifications tested for the VIS1-HS method and resulting mean total tumor cell count and Ki67 score.

Specifications (VIS1)			N=	Mean total tumor cell count	%Ki67		
Drawing radius	Size of ROI	Number of ROIs			Mean	Median	Range
**175µm**	0.2mm^2	1	98	705	18.0	13.8	0.8 – 87.8
**400µm**	0.2mm^2	1	98	704	16.7	10.7	1.0 – 76.9
**175µm**	1mm^2	1	98	2849	15.7	10.6	1.0 – 77.4
**400µm**	1mm^2	1	98	3360	16.0	10.9	0.4 – 81.1
400µm	0.2mm^2	**2**	27	782	22.3	16.1	4.7 – 68.2
400µm	0.2mm^2	**3**	27	752	22.0	17.1	4.8 – 64.6
400µm	0.2mm^2	**4**	26	753	20.2	16.4	4.4 – 62.4
400µm	0.2mm^2	**5**	26	753	20.3	15.6	4.5 – 60.1
400µm	**550 TC min**	1	27	692	23.2	17.5	4.2 – 74.9
400µm	**1000 TC min**	1	27	1119	23.0	16.2	3.7 – 76.2

These specifications were used for the main investigations of the study.

Abbreviations: ROI = region of interest, TC = total tumor cell count, VIS1-HS = Visiopharm in-house hotspot

Supplementary Table S6

Supplementary table S6: Performance of tested cutoffs for binary categorization of Ki67 score as assessed by manual and digital image analysis scoring methods.

**Method (Ki67 cut-off)**	Log-rank (p-value)	N= False negative	False positive	True negative	True positive	Sens.	Spec.
Conventional							
3.5%	0.004	4	153	75	35	88%	32%
6.5%	0.001	9	114	114	30	76%	49%
10.6%	0.001	14	83	145	25	63%	64%
**14%**	**0.026**	**20**	**67**	**161**	**19**	**49%**	**71%**
20%	0.023	25	46	182	14	36%	80%
30%	0.004	29	24	204	10	26%	89%
48%	0.253	35	11	217	4	10%	95%
Global UW							
2%	0.068	4	195	59	36	92%	23%
8%	0.031	12	134	120	28	73%	49%
10%	0.016	13	121	133	27	70%	53%
**14%**	**0.021**	**19**	**86**	**168**	**21**	**51%**	**68%**
20%	0.317	26	68	186	14	38%	74%
31%	0.087	29	40	214	11	30%	84%
40%	0.011	32	21	233	8	22%	92%
Global W							
2%	0.110	5	193	61	35	89%	24%
5%	0.069	10	153	101	30	76%	40%
10%	0.007	13	113	141	27	70%	56%
**14%**	**0.020**	**19**	**86**	**168**	**21**	**57%**	**67%**
20%	0.081	25	61	193	15	41%	77%
30%	0.198	30	41	213	10	27%	84%
40%	0.008	31	23	231	9	24%	90%
VIS1-HS							
4%	0.022	2	198	56	38	95%	22%
8%	0.001	9	126	128	31	78%	50%
10%	0.001	12	103	151	28	70%	59%
**14%**	**0.000**	**18**	**67**	**187**	**22**	**55%**	**74%**
20%	0.000	23	46	208	17	43%	82%
30%	0.002	31	21	233	9	23%	92%
41%	0.858	38	12	242	2	5%	95%
VIS2-HS							
3.4%	0.005	1	195	59	39	98%	23%
6%	0.001	5	149	105	35	88%	41%
10%	0.000	11	106	148	29	73%	58%
**14%**	**0.000**	**16**	**75**	**179**	**24**	**60%**	**70%**
20%	0.007	23	57	197	17	43%	78%
30%	0.001	29	27	227	11	28%	89%
41%	0.136	35	15	239	5	13%	94%
VIS2-G							
2.3%	0.027	5	178	76	35	81%	28%
7.6%	0.001	15	86	168	25	62%	68%
10%	0.001	19	67	187	21	46%	75%
**14%**	**0.000**	**22**	**48**	**206**	**18**	**45%**	**81%**
20%	0.010	30	28	226	10	25%	89%
42%	0.190	37	8	246	3	12%	98%
QuPath							
**14%**	**0.000**	**12**	**82**	**163**	**28**	**70%**	**67%**
20%	0.000	19	62	183	21	53%	75%
30%	0.000	24	33	212	16	40%	87%

Abbreviations: Sens. = sensitivity, Spec. = specificity, UW = unweighted, W = weighted, VIS1-HS = Visiopharm in-house hotspot, VIS2-HS = Visiopharm CE-IVD hotspot, VIS2-G = Visiopharm CE-IVD global.

Supplementary Table S7

Supplementary table S7: Percentage agreement of Ki67 quantification methods using a 14% cutoff.

**Method**	**Measure**	**Method**					
		Conventional	*Global uW*	*Global W*	*VIS1-HS*	*VIS2-HS*	*VIS2-G*
Conventional	% Agreement						
	% Disagreement						
Global uW	% Agreement	87%					
	% Disagreement	13%					
Global W	% Agreement	85%					
	% Disagreement	15%					
VIS1-HS	% Agreement	91%	86%	84%			
	% Disagreement	9%	14%	16%			
VIS2-HS	% Agreement	91%	89%	88%	95%		
	% Disagreement	9%	11%	12%	5%		
VIS2-G	% Agreement	89%	84%	84%	89%	88%	
	% Disagreement	11%	16%	16%	11%	12%	
QuPath	% Agreement	87%	87%	86%	89%	92%	83%
	% Disagreement	13%	13%	14%	11%	8%	17%

Abbreviations: uW = unweighted, W = weighted, VIS1-HS = Visiopharm in-house hotspot, VIS2-HS = Visiopharm CE-IVD hotspot, VIS2-G = Visiopharm CE-IVD global.

Supplementary Fig. S1

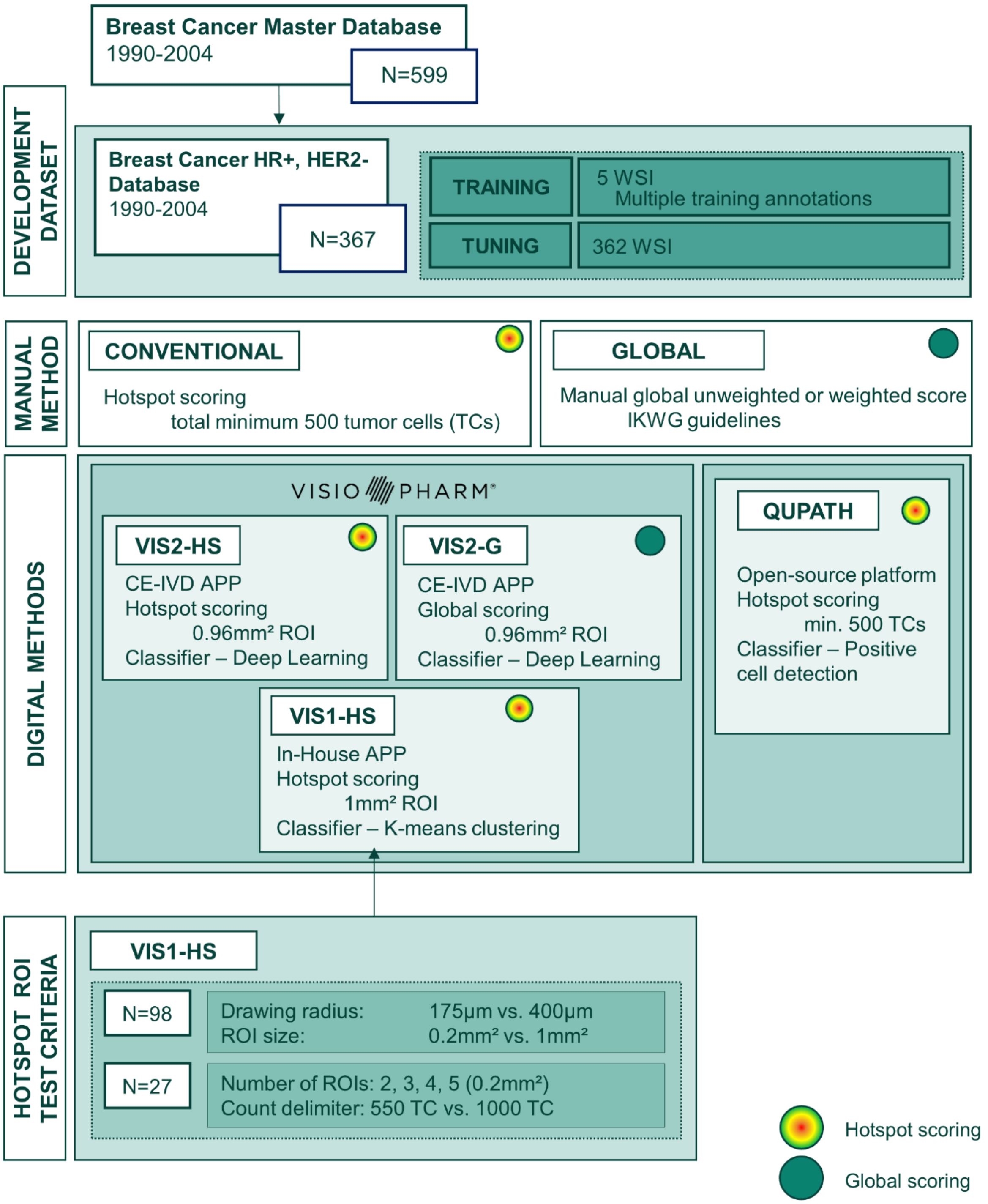

Fig. S1 Case summary of the study cohort. A development dataset was used to train and tune two in-house Ki67 scoring algorithms using a commercial platform (VIS1-HS) and an open-source platform (QuPath). Cases from the development dataset were also evaluated using a using two manual methods (conventional hotspot Ki67 scoring and global unweighted and weighted scoring) and a commercial CE-IVD application (VIS2-HS/G). Specifications for each method is outlined. A variety of parameters was investigated for specification of hotspot ROI criteria. *Abbreviations: HR = hormone receptor, WSI = whole slide image, TC = total tumor cell count, IKWG = International Ki67 in Breast Cancer Working Group, HS = hotspot, G = global, APP = application, ROI = region of interest, HS = hotspot, VIS1-HS = Visiopharm in-house hotspot, VIS2-HS = Visiopharm CE-IVD hotspot, VIS2-G = Visiopharm CE-IVD global.*

Supplementary Fig. S2

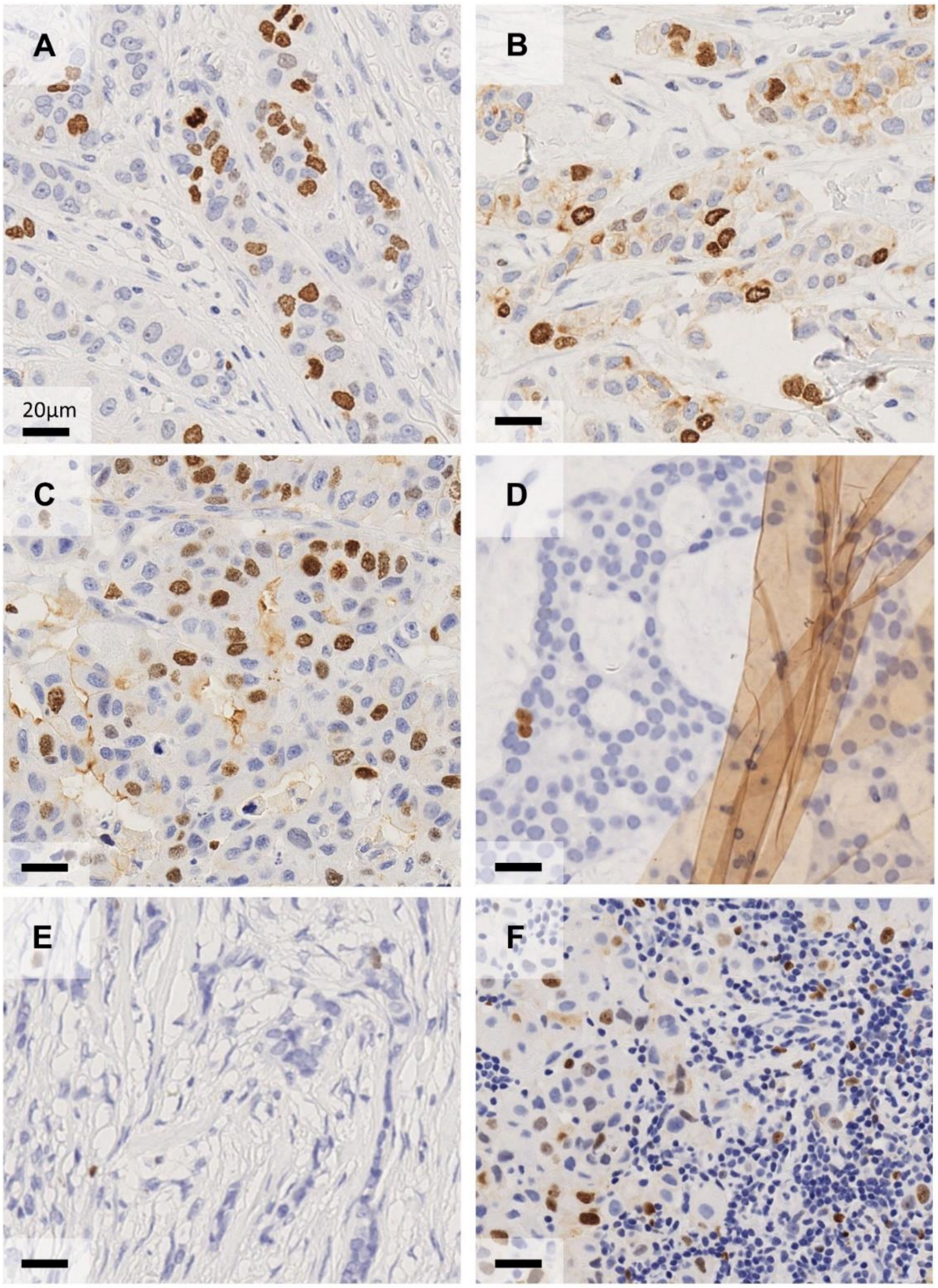

Fig. S2 Examples of optimal and poor quality Ki67 staining patterns and artefacts. A) Optimal Ki67 staining for clear distinction of negative (blue) nuclei and positive (brown) nuclei. B-C) Background staining observed in the cytoplasm and a staining edge effect observed between tumor cell clusters. This affected analysis to varying degrees depending on the intensity of the background stain, software used and area coverage (smaller areas could be excluded from analysis). A case was rejected when background staining resulted in an unacceptable number of false positive nuclei detections. D) Example of an artefact that required manual removal due to the negative (blue nuclei) beneath the fragment being labelled as positive by all digital methods. E) An example of a poor quality tissue section likely due to poor fixation. F) A region where a high number of tumor infiltrating lymphocytes are present amongst tumor cells. These regions could cause the analysis to be rejected or were manually excluded from analysis.

Supplementary Fig. S3

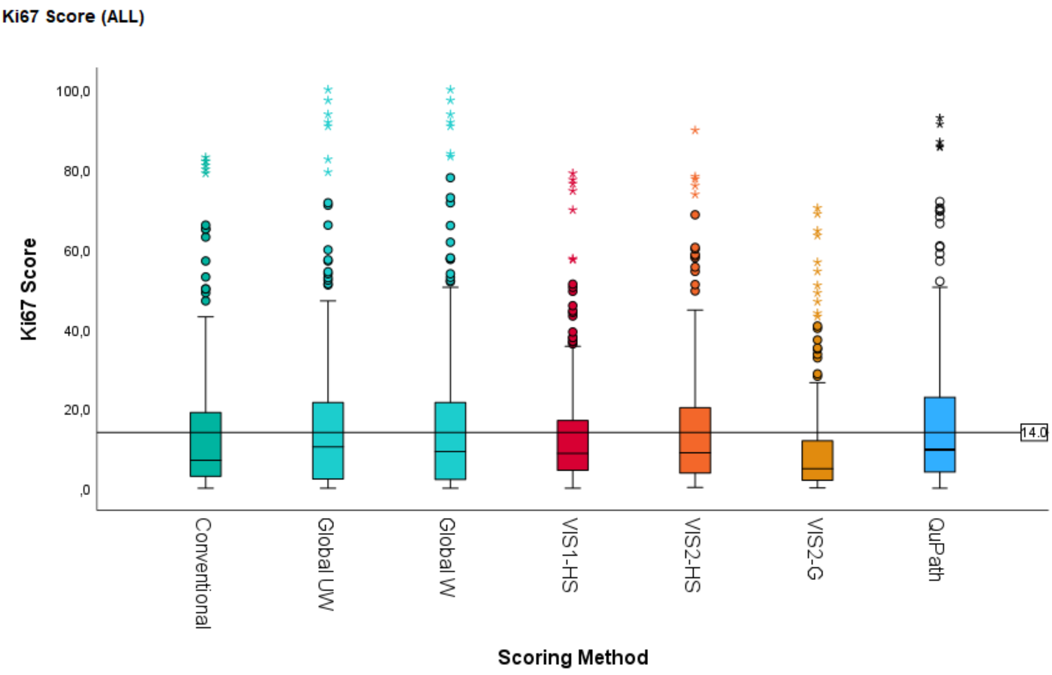

Fig. S3 The distribution of Ki67 score for all manual (conventional-HS, global unweighted-UW and weighted-W) and automated (VIS1-HS, VIS2-HS, VIS2-G, QuPath) scoring methods. The line intersecting all box plots indicates 14%. *Abbreviations: UW = unweighted, W = weighted, VIS1-HS = Visiopharm in-house hotspot, VIS2-HS = Visiopharm CE-IVD hotspot, VIS2-G = Visiopharm CE-IVD global.*

Supplementary Fig. S4

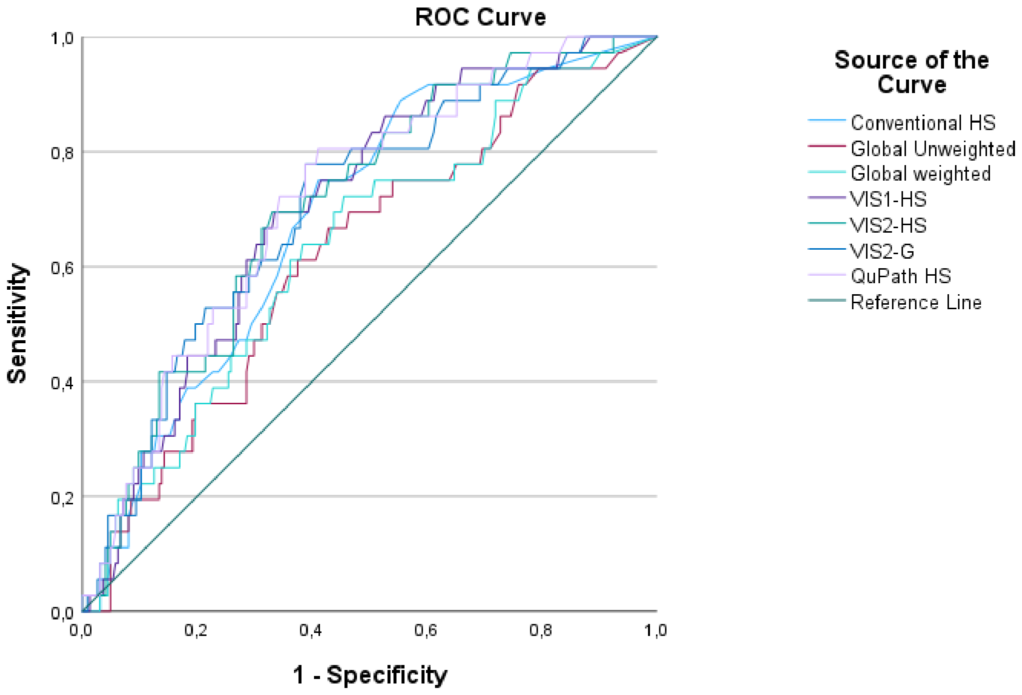

Fig. S4 ROC curves for manual (conventional HS, global weighted, global unweighted) and digital (VIS1-HS, VIS2-HS, VIS2-G, QuPath HS) Ki67 scoring methods. *Abbreviations: HS = hotspot, VIS1-HS = Visiopharm in-house hotspot, VIS2-HS = Visiopharm CE-IVD hotspot, VIS2-G = Visiopharm CE-IVD global.*
